# Hepatic leukemia factor-expressing paraxial mesoderm cells contribute to the developing brain vasculature

**DOI:** 10.1242/bio.059510

**Published:** 2022-09-15

**Authors:** Yuta Koui, Takako Ideue, Michael Boylan, Matthew J. Anderson, Motomi Osato, Toshio Suda, Tomomasa Yokomizo, Yoh-suke Mukouyama

**Affiliations:** 1 Laboratory of Stem Cell and Neuro-Vascular Biology, Cell and Development Biology Center, National Heart, Lung, and Blood Institute, National Institutes of Health, Bethesda, MD 20892, USA; 2 International Research Center for Medical Sciences, Kumamoto University, Kumamoto 860-0811, Japan; 3 Cancer and Developmental Biology Laboratory, National Cancer Institute, National Institutes of Health, Frederick, MD 21702, USA; 4 Cancer Science Institute of Singapore, National University of Singapore, Singapore 117599; 5 Department of Microscopic and Developmental Anatomy, Tokyo Women's Medical University, Tokyo 162-8666, Japan

**Keywords:** Hepatic leukemia factor, Cephalic mesenchyme, Lineage tracing, Endothelial cells, Pericytes

## Abstract

Recent genetic lineage tracing studies reveal heterogeneous origins of vascular endothelial cells and pericytes in the developing brain vasculature, despite classical experimental evidence for a mesodermal origin. Here we provide evidence through a genetic lineage tracing experiment that cephalic paraxial mesodermal cells give rise to endothelial cells and pericytes in the developing mouse brain. We show that *Hepatic leukemia factor* (*Hlf*) is transiently expressed by cephalic paraxial mesenchyme at embryonic day (E) 8.0-9.0 and the genetically marked E8.0 Hlf-expressing cells mainly contribute to the developing brain vasculature. Interestingly, the genetically marked E10.5 Hlf-expressing cells, which have been previously reported to contain embryonic hematopoietic stem cells, fail to contribute to the vascular cells. Combined, our genetic lineage tracing data demonstrate that a transient expression of *Hlf* marks a cephalic paraxial mesenchyme contributing to the developing brain vasculature.

This article has an associated First Person interview with the first author of the paper.

## Abstract (precis)

**Summary:** Genetic lineage tracing reveals that a transient expression of hepatic leukemia factor (Hlf) marks angiogenic cephalic paraxial mesenchyme, which mainly contributes to the developing brain vasculature.

## INTRODUCTION

During vertebrate development, the segmented paraxial mesoderm of the somite gives rise to different mesodermal derivatives including vascular cells. The quail-chicken chimera and labeling system demonstrated that vascular progenitors derive from the somite and contribute to the trunk (Pardanaud et al., 1996; Pouget et al., 2006; Sato et al., 2008) and limb vasculature (Huang et al., 2003; Kardon et al., 2002). Genetic lineage tracing experiments with the *Cre-loxP* system clearly demonstrated the contribution of the somatic vascular progenitors into the trunk and limb vasculature: paraxial mesoderm-specific *Cre* lines such as *Meox1-Cre*, *Pax3-Cre*, and *Myf5-Cre* showed that paraxial mesodermal cells contribute to the trunk and limb vasculature (Hutcheson et al., 2009; Mayeuf-Louchart et al., 2014; Stone and Stainier, 2019; Wasteson et al., 2008). What induces paraxial mesodermal cells to differentiate into tissue-specific vascular cells remains to be investigated.

Previous studies with the quail-chicken chimera and labeling system demonstrated that the cephalic paraxial mesoderm has an angiogenic potential and contributes to the head and neck vasculature (Couly et al., 1995). In the developing brain and spinal cord of the central nervous system (CNS), the cephalic paraxial mesoderm-derived angioblasts are assembled to form perineural vascular plexus (PNVP) around the neural tube (Hogan et al., 2004; Jukkola et al., 2005). Subsequently, sprouting vessels from the PNVP invade the CNS tissues and extend the branches from the plexus towards the ventricle (Gupta et al., 2021; Paredes et al., 2018; Tata et al., 2015). In addition to the mesodermal origin, recent genetic lineage tracing studies with the *Cre-loxP* system demonstrated that erythro-myeloid progenitors (EMPs) contribute to brain endothelial cells (Plein et al., 2018), although contradictory data were reported (Feng et al., 2020; Palis and Yoder, 2020). The cephalic neural crest cells penetrate and differentiate into pericytes in the forebrain vasculature (Korn et al., 2002; Reyahi et al., 2015; Yamanishi et al., 2012). Genetic lineage tracing studies have revealed heterogeneous origins of CNS vascular cells from distinct populations, but CNS vascular cells derived via a classical pathway of mesodermal differentiation into endothelial cells and pericytes were not examined.

Here, we studied the developmental timing of angiogenic cephalic paraxial mesenchyme in the developing vasculature of the CNS and various tissues using the *Cre-loxP*-based lineage tracing system. First, we found a unique expression of hepatic leukemia factor (Hlf), the proline and acid rich basic region leucine zipper (Par-bZip) transcription factor, in the cephalic paraxial mesenchyme at embryonic day (E) 8.5. Second, the lineage tracing experiments using *Hlf-Cre^ERT2^; ROSA-LSL-tdTomato* embryos, with tamoxifen administration at E8.0, revealed that a transient expression of *Hlf* marked angiogenic cephalic mesenchyme, which mainly contributes to vascular cells in the developing CNS tissues at E15.5. Interestingly, a transient expression of *Hlf* at E10.5 failed to mark vascular cells at E15.5, suggesting that *Hlf* marks an angiogenic paraxial mesenchyme subpopulation from an early stage in vascular development.

## RESULTS

### Hlf-expressing cells in the cephalic mesoderm but not yolk sac

**Fig. 1.**
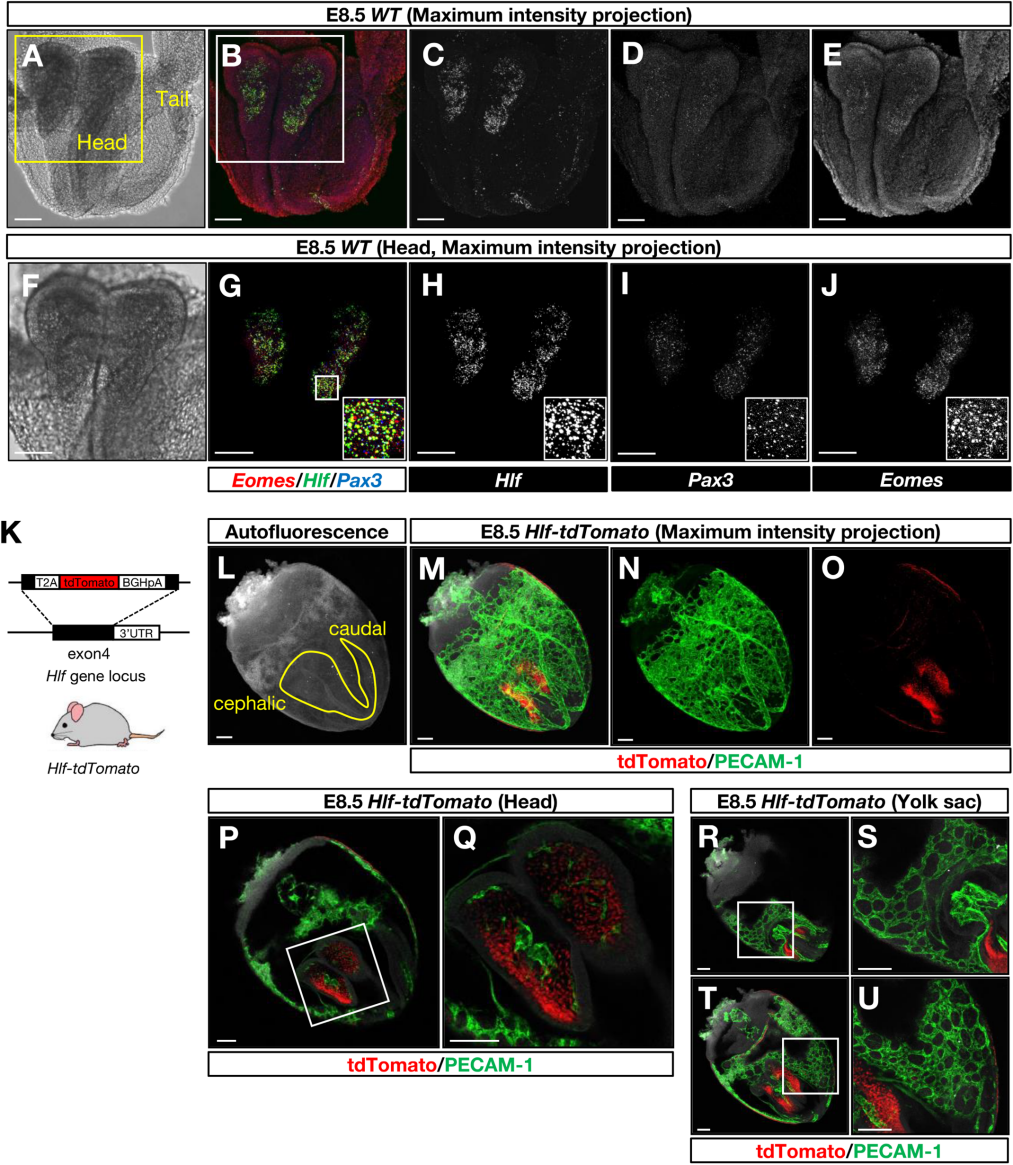
**Hlf-expressing cells in the cephalic mesenchyme at E8.5.** (A-J) Triple RNA whole-mount *in situ* hybridization chain reaction of E8.5 wild-type (WT) embryos. (A,F) The position of E8.5 embryos is indicated on the bright field image. The boxed region in A is magnified in F. Scale bars: 100 µm. (B-E,G-J) Maximum intensity projection images from triple RNA whole-mount *in situ* hybridization chain reaction using probes to *Hlf* (B and G, green; C and H, white) and the paraxial mesoderm marker *Pax3* (B and G, blue; D and I, white) and *Eomes* (B and G, red; E and J, white). The boxed region in B is magnified in G. The boxed region in G is magnified in the bottom right. Scale bars: 100 µm. (K) Generation of *Hlf-tdTomato* reporter mice (Yokomizo et al., 2019). (L) The position of E8.5 embryo is indicated on the autofluorescence image of 488-channel. Scale bars: 100 µm. (M-O) Maximum intensity projection images from whole-mount immunofluorescent labeling of E8.5 *Hlf-tdTomato* reporter embryos using antibodies to tdTomato (red) and the pan-endothelial cell marker PECAM-1 (green). Scale bars: 100 µm. *n*=2. (P-U) Representative Z-slice images from whole-mount immunofluorescent labeling of E8.5 *Hlf-tdTomato* reporter embryos. The boxed regions in P, R and T are magnified in Q, S and U, respectively. Scale bars: 100 µm. *n*=2.

Previous studies revealed that *Hlf* expression marks embryonic hematopoietic stem cell (HSC) precursors within the dorsal aorta of the aorta-gonad-mesonephros (AGM) region at E10.5 and maturing HSCs in the fetal liver between E11.5 and E14.5 (Yokomizo et al., 2019). Moreover, *Hlf* expression does not mark EMPs within the yolk sac or endothelial cells of the yolk sac, AGM, or fetal liver (Yokomizo et al., 2019). These observations were supported by the recent report by Tang et al. (2021). Compared with the unique *Hlf* expression in embryonic HSCs from E10.5 to E14.5, *Hlf* expression in non-hematopoietic cells remains elusive. To address this, we initially analyzed the published single cell RNA-sequence data set of E8.0 and E8.5 mouse embryo (Pijuan-Sala et al., 2019). At these stages, most hematopoietic progenitors emerge in the yolk sac and migrate to intra-embryonic organs via circulation (Gomez Perdiguero et al., 2015; Hoeffel et al., 2015; Lux et al., 2008). Indeed, the clusters of blood progenitors and erythrocytes express RUNX family transcription factor 1 (*Runx1*), which is known to be expressed by hematopoietic progenitors and HSCs (Utz et al., 2020), while few blood progenitors and erythrocytes express *Hlf* (Fig. S1A-F). In contrast, *Hlf* expression is highly enriched in the paraxial mesoderm in both E8.0 and E8.5 embryos (Fig. S1A-F). To confirm the expression pattern of *Hlf* in E8.5 embryos, we first performed RNA whole-mount *in situ* hybridization chain reaction (HCR). *Hlf* expression was clearly detectable in the cephalic region, where the angiogenic cephalic mesenchyme was found in the quail-chicken chimera and labeling experiment (Couly et al., 1995) (Fig. 1A-J). Most of *Hlf*-expressing cells express the paraxial mesoderm markers *Pax3* or *Eomes* or both (Fig. 1A-J), indicating that these *Hlf*-expressing cells are cephalic paraxial mesodermal cells. These findings were consistent with the published single cell RNA-sequence data set (Pijuan-Sala et al., 2019). We further performed the whole-mount immunohistochemical analysis of *Hlf-tdTomato* knock-in reporter embryos at E8.5 (Fig. 1K-U). Hlf-tdTomato-expressing cells were clearly detectable in the cephalic region (Fig. 1L-O; Fig. S2A-H), but these cells are negative for the endothelial cell marker PECAM-1 in the head or the yolk sac vasculature at E8.5 (Fig. 1P and Q, head; Fig. 1R-U, yolk sac). Combined, these data suggest that Hlf-expressing cells at E8.5 represent a mesenchyme subpopulation in the cephalic region.

### Transient expression of Hlf marked cephalic mesenchyme

**Fig. 2.**
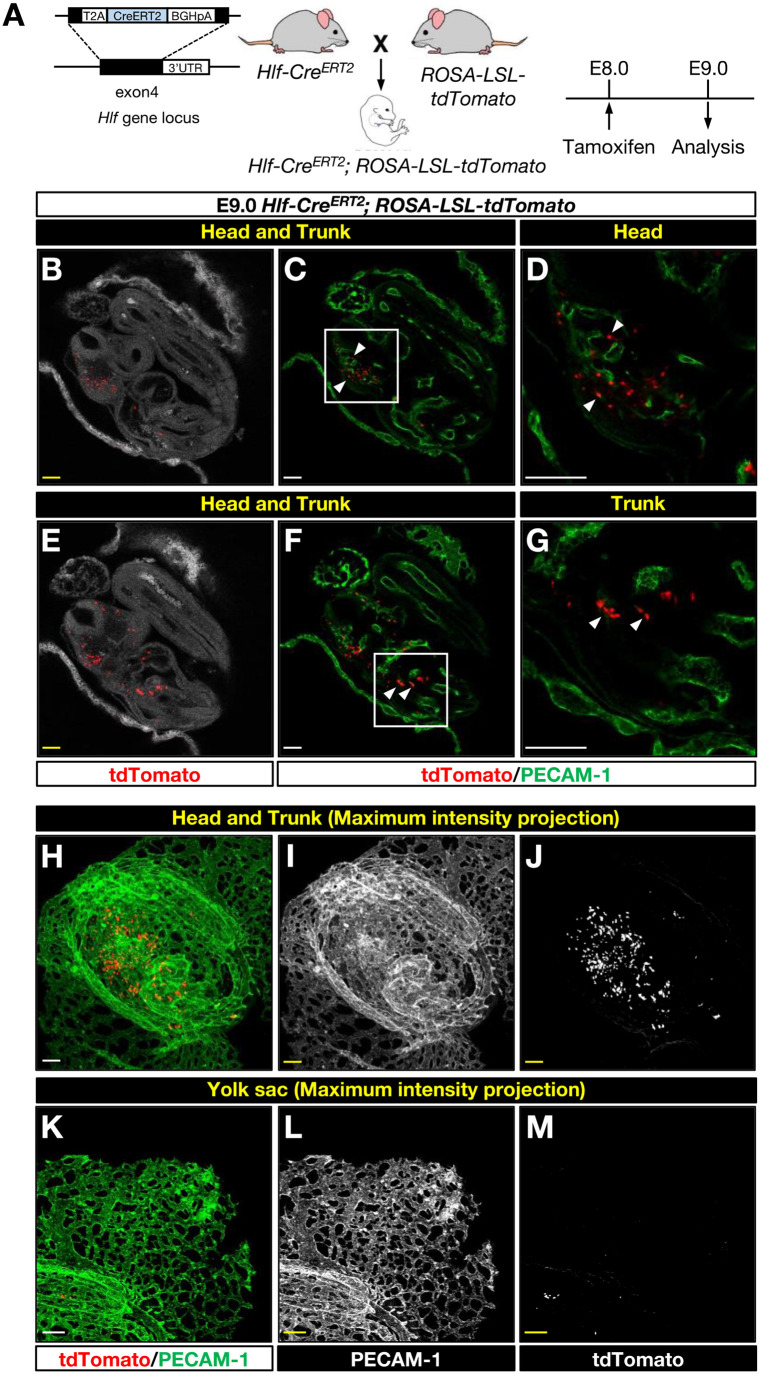
**Transient expression of Hlf marks cephalic mesenchymal cells.** (A) Generation of *Hlf-Cre^ERT2^* mice and *Hlf-Cre^ERT2^; ROSA-LSL-tdTomato* embryos. Tamoxifen was administered by oral gavage at E8.0 and embryos were harvested at E9.0 for analysis. (B-G) Representative Z-slice images from whole-mount immunofluorescent labeling of E9.0 *Hlf-Cre^ERT2^; ROSA-LSL-tdTomato* embryos with antibodies to tdTomato (red) and PECAM-1 (green). The boxed regions in C and F are magnified in D and G, respectively. Arrowheads indicate tdTomato-expressing Hlf-lineage cells. Scale bars: 100 µm. *n*=6. (H-M) Maximum intensity projection images from whole-mount immunofluorescent labeling of *Hlf-Cre^ERT2^; ROSA-LSL-tdTomato* embryos at E9.0 with antibodies to tdTomato (H and K, red; J and M, white) and PECAM-1 (H and K, green; I and L, white). Scale bars: 100 µm. *n*=6.

We next examined differentiation potentials of Hlf-expressing cephalic mesenchymal cells using a tamoxifen-induced *Hlf-Cre^ERT2^* knock-in mice in combination with a *Cre*-mediated *ROSA-LSL-tdTomato* reporter mice (Fig. 2A). Like *Hlf-tdTomato* knock-in reporter mice (Yokomizo et al., 2019), *Hlf-Cre^ERT2^* mice were generated by inserting a *T2A-Cre^ERT2^* gene fusion before the endogenous stop codon within exon 4 (Fig. 2A). We first induced tdTomato expression in *Hlf-Cre^ERT2^; ROSA-LSL-tdTomato* embryos at E8.0 and used high-resolution whole-mount imaging to analyze the distribution of tdTomato-expressing Hlf-lineage cells at E9.0 (Fig. 2A). Double RNA whole-mount *in situ* HCR using probes to *Hlf* and *tdTomato* revealed that *Hlf* and *tdTomato* signals have been largely overlapping in the cephalic region (Fig. S2I-P). Consistent with the analysis of *Hlf-tdTomato* reporter embryos at E8.5, we found the majority of Hlf-lineage cells were detectable in the cephalic region (Fig. 2B-G; Fig. S2I-P): a few Hlf-lineage cells were found in the rostral trunk region (Fig. 2B-G). Hlf-lineage cells were not detectable in the head, trunk, and yolk sac vasculature at E9.0 (Fig. 2C,D,F,G,H,I and J, head and trunk; K, L, and M, yolk sac). These data suggest that most Hlf-expressing cells at E8.0-9.0 represent cephalic mesenchymal cells.

### Hlf-expressing cephalic mesenchymal cells contribute to the brain vasculature

**Fig. 3.**
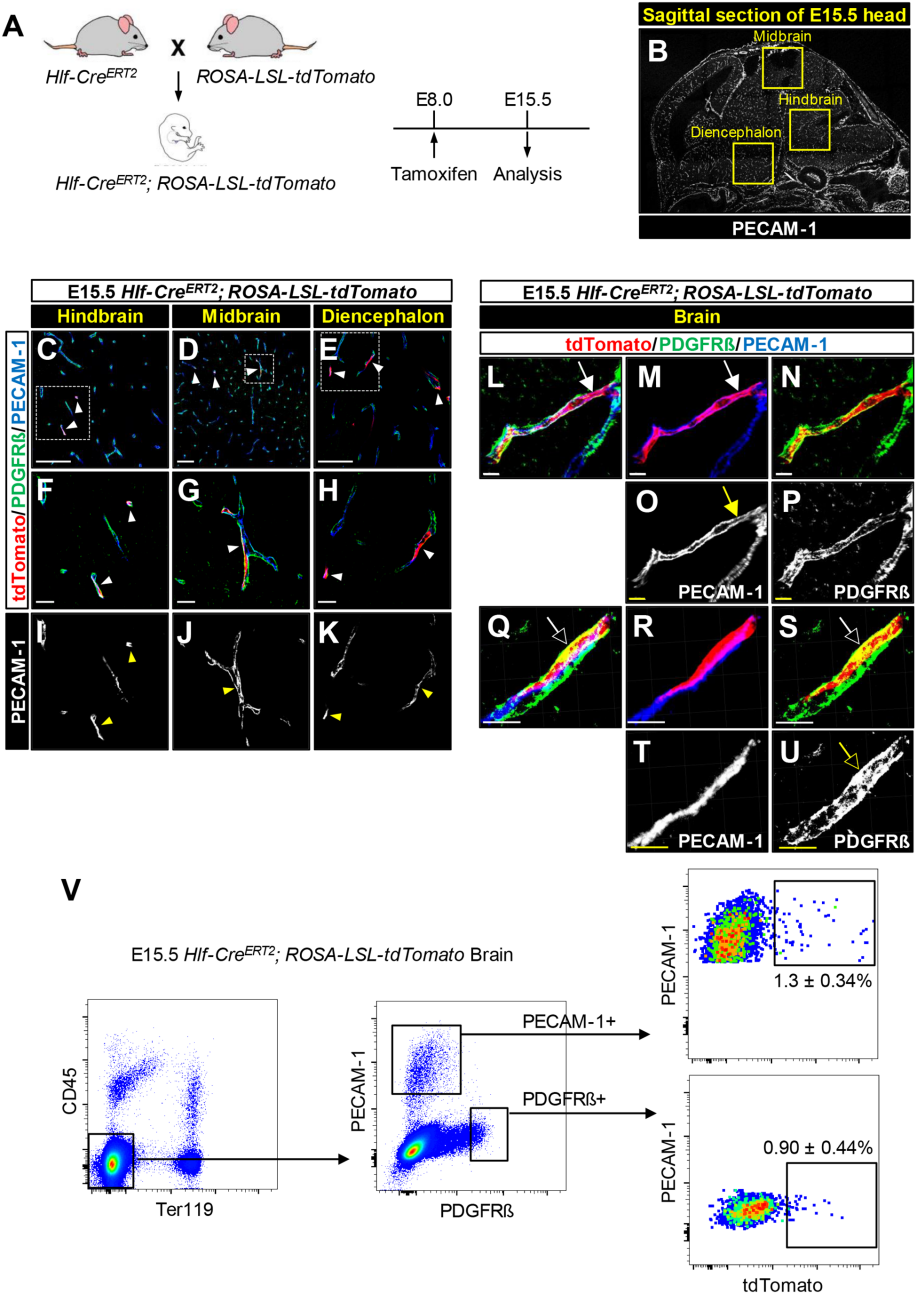
**Hlf-expressing cephalic mesenchymal cells contribute to the brain vasculature.** (A) Generation of *Hlf-Cre^ERT2^; ROSA-LSL-tdTomato* embryos. Tamoxifen was administered by oral gavage at E8.0 and embryos were harvested at E15.5 for analysis. (B) A representative immunofluorescent image of sagittal sections of E15.5 head stained with anti-PECAM-1 antibody (white). The yellow boxes indicate hindbrain, midbrain, and diencephalon in the brain parenchyma. (C-K) Representative immunofluorescent images of E15.5 *Hlf-Cre^ERT2^; ROSA-LSL-tdTomato* brain sections stained with antibodies to tdTomato (C-H, red) together with the pericyte marker PDGFRß (C-H, green) and the endothelial cell marker PECAM-1 (C-H, blue; I-K, white). The boxed regions in (C-E) are magnified in (F-K). Arrowheads indicate tdTomato^+^ vascular cells. Note that quantification of tdTomato^+^ blood vessels in the hindbrain, midbrain, and diencephalon is shown in Fig. 4R. Scale bars: 100 µm (C-E) and 20 µm (F-K). *n*=3. (L-U) High-resolution images of E15.5 *Hlf-Cre^ERT2^; ROSA-LSL-tdTomato* brain sections stained with antibodies to tdTomato (red) together with PDGFRß (L, N, Q, and S, green; P and U, white) and PECAM-1 (L, M, Q, and R, blue; O and T, white). Arrows indicate tdTomato^+^/PECAM-1^+^ endothelial cells; open arrows indicate tdTomato^+^/PDGFRß^+^ pericytes. Scale bars: 10 µm. *n*=3. (V) Representative flow cytometry data analyzing E15.5 *Hlf-Cre^ERT2^; ROSA-LSL-tdTomato* brain. Non-hematopoietic cells are negative for the pan-hematopoietic marker CD45 and erythrocyte marker Ter119. Endothelial cells are positive for PECAM-1 and pericytes are positive for PDGFRß. Positive gates were defined by the non-staining controls. Percentages of tdTomato^+^ cells in the fractions of endothelial cells (CD45^−^/Ter119^−^/PECAM-1^+^/PDGFRß^−^) and pericytes (CD45^−^/Ter119^−^/PECAM-1^−^/PDGFRß^+^) were calculated. *n*=3.

**Fig. 4.**
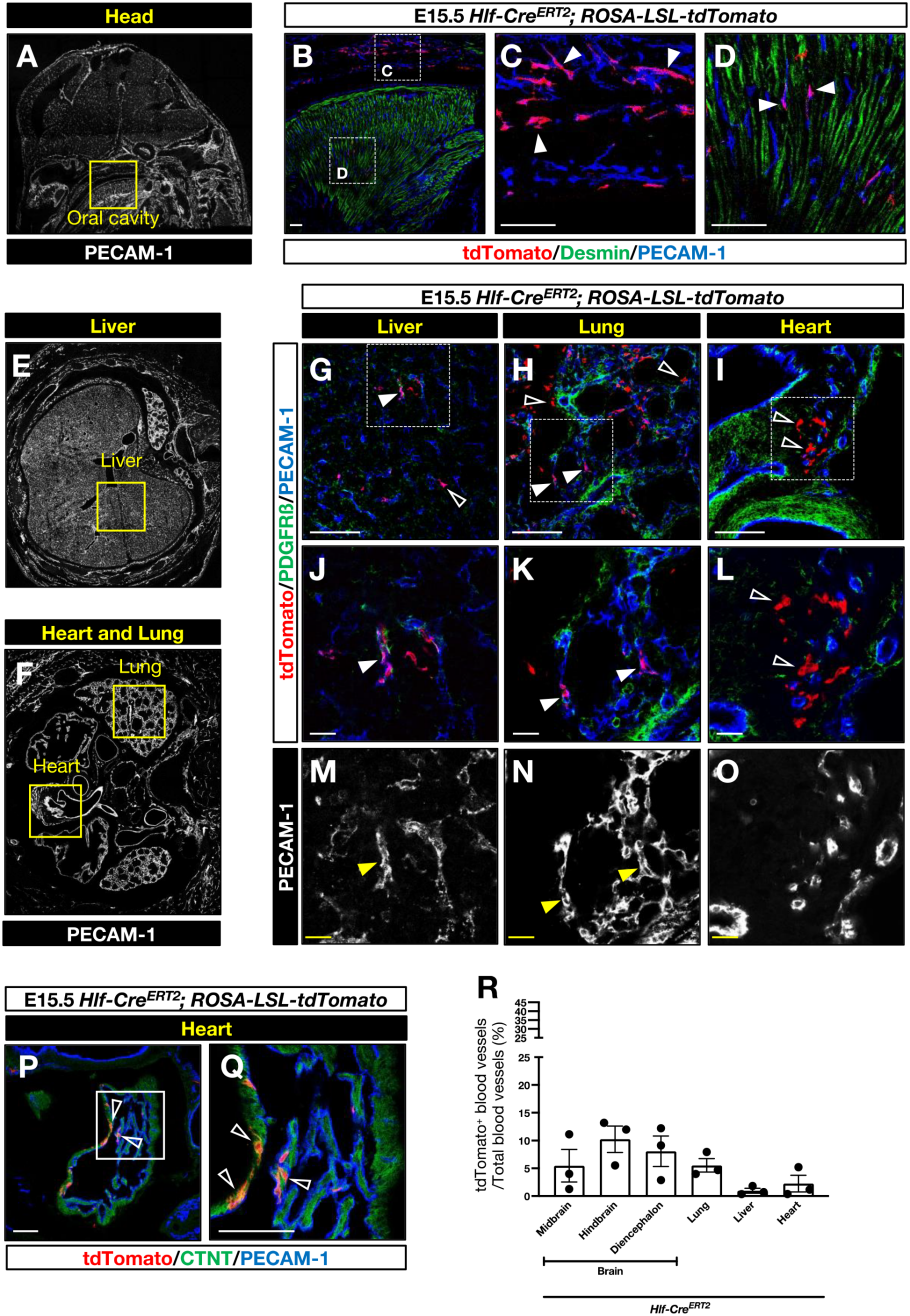
**Contribution of Hlf-expressing cells to the vasculature of head, liver, lung, and heart.** (A) A representative immunofluorescent image of sagittal sections of E15.5 head stained with anti-PECAM-1 antibody (white). The yellow box indicates oral cavity. (B-D) Representative immunofluorescent images of oral cavity sections from E15.5 *Hlf-Cre^ERT2^; ROSA-LSL-tdTomato* stained with antibodies to tdTomato (red) together with the muscle progenitor marker Desmin (green) and the endothelial marker PECAM-1 (blue). The boxed regions in B are magnified in C and D. Arrowheads indicate tdTomato^+^ vascular cells. Scale bars: 100 µm. *n*=3. (E,F) A representative immunofluorescent image of transverse sections of the trunk region of E15.5 mouse embryos including liver (E), lung (F), and heart (F) stained with anti-PECAM-1 antibody (white). The yellow boxes show the region of liver, lung, and heart in the trunk region. (G-O) Representative immunofluorescent images of liver, lung and heart sections of E15.5 *Hlf-Cre^ERT2^; ROSA-LSL-tdTomato* embryos stained with antibodies to tdTomato (red), PDGFRß (green) and PECAM-1 (G-L, blue; M-O, white). The boxed regions in G-I are magnified in J-O. Arrowheads indicate tdTomato^+^ vascular cells; open arrowheads indicate tdTomato^+^ non-vascular cells. Scale bars: 100 µm (G-I) and 20 µm (J-O). *n*=3. (P-Q) Representative immunofluorescent images of heart sections of E15.5 *Hlf-Cre^ERT2^; ROSA-LSL-tdTomato* embryos stained with antibodies to tdTomato (red), cardiac troponin T (CTNT, green) and PECAM-1 (blue). The boxed region in P is magnified in Q. Open arrowheads indicate tdTomato^+^ cardiomyocytes. (R) Quantification of tdTomato^+^ blood vessels in each tissue. The results are shown as the mean±s.e.m. *n*=3 in each group.

We next examined the contribution of Hlf-expressing cephalic mesenchymal cells to the developing brain vasculature. In addition to the *Hlf-Cre^ERT2^; ROSA-LSL-tdTomato* lineage tracing mouse model, we used a tamoxifen-inducible *Runx1-Cre^ERT2^; ROSA-LSL-tdTomato* lineage tracing mouse model as a reference for the lineage tracing experiments in both vascular and hematopoietic cells: *Runx1* is known to be expressed by endothelial cells as well as HSCs and hematopoietic progenitors including the yolk sac EMPs at E8.0 (Hoeffel et al., 2015; Ng et al., 2010; Samokhvalov et al., 2007). *Runx1-Cre^ERT2^* transgenic mice were generated by introducing multiple copies of the Runx1 enhancer element eR1-driven *CreERT2* cassette (Matsuo et al., 2017). We induced *tdTomato* expression in *Hlf-Cre^ERT2^; ROSA-LSL-tdTomato* and *Runx1-Cre^ERT2^; ROSA-LSL-tdTomato* embryos at E8.0 and analyzed the distribution of tdTomato-expressing Hlf- and Runx1-lineage cells in the developing brain parenchyma such as hindbrain, midbrain, and diencephalon at E15.5 (Fig. 3A,B; Fig. S3A). Interestingly, all tdTomato-expressing Hlf-lineage cells were found in the developing vasculature of the hindbrain (10.2±2.4% of blood vessels were positive for tdTomato), the midbrain (5.5±2.3%), and the diencephalon (8.1±2.8%) of *Hlf-Cre^ERT2^; ROSA-LSL-tdTomato* brain at E15.5 (Fig. 3C-K, arrowheads; Fig. 4R). Hlf-lineage cells were rarely found in the cerebral cortex. High-resolution imaging revealed the tdTomato expression in PECAM-1^+^ endothelial cells as well as PDGFRß^+^ pericytes (Fig. 3L-P, arrows, tdTomato^+^/PECAM-1^+^ endothelial cells; 3Q-U, open arrows, tdTomato^+^/ PDGFRß^+^ pericytes). Flow cytometric analysis of *Hlf-Cre^ERT2^; ROSA-LSL-tdTomato* brain at E15.5 also revealed that Hlf-lineage cells contribute to both endothelial cells and pericytes in the developing brain vasculature (Fig. 3V, 1.3±0.34% of endothelial cells from whole-brain were positive for tdTomato and 0.90±0.44% of pericytes from whole-brain were positive for tdTomato). These results clearly demonstrate that cephalic mesenchyme contributes to the developing brain vasculature. In contrast, tdTomato-expressing Runx1-lineage cells contribute to both vascular cells and non-vascular cells in the brain parenchyma (Fig. S3B-J, arrowhead, vascular cells; open arrowheads, non-vascular cells; Fig. S4M) such as the hindbrain (18.1±5.8% of blood vessels were positive for tdTomato), the midbrain (7.4±2.3%), and the diencephalon (8.9±1.4%) of E15.5 *Runx1-Cre^ERT2^; ROSA-LSL-tdTomato* brain (Fig. S4M). Like *Hlf-Cre^ERT2^; ROSA-LSL-tdTomato* brain, we found tdTomato expression in PECAM-1^+^ endothelial cells as well as PDGFRß^+^ pericytes (Fig. S3K-O, arrows, tdTomato^+^/PECAM-1^+^ endothelial cells; S3P-T, open arrows, tdTomato^+^/PDGFRß^+^ pericytes). Both Hlf- and Runx1-lineage cells contribute to the developing vasculature in three major regions of the brain parenchyma, albeit with different levels of contribution to vascular cells (Fig. 4R versus Fig. S4M). All these results indicate that, compared with Runx1-lineage cells, the differentiation potential of Hlf-expressing cephalic mesenchymal cells at E8.0 appears to be restricted to the developing brain vasculature.

### No contribution of Hlf-expressing cephalic mesenchymal cells to the muscular tissues

We next examined a contribution of Hlf-lineage cells to muscular tissues in the embryonic head such as tongue in the oral cavity (Fig. 4A). Given that tdTomato-expressing Hlf-lineage cells were found in PECAM-1^+^ vasculature in the tongue of *Hlf-Cre^ERT2^; ROSA-LSL-tdTomato* embryos at E15.5, Hlf-lineage cells were not detectable in muscle progenitors which express Desmin, a muscle-specific intermediate filament (Fig. 4B-D). Likewise, tdTomato-expressing Runx1-lineage cells were found in PECAM-1^+^ vasculature but not in Desmin^+^ muscle progenitors in the tongue of E15.5 *Runx1-Cre^ERT2^; ROSA-LSL-tdTomato* embryos (Fig. S4A-C). These data suggest that Hlf-expressing cells at E8.0 are a unique subset of cephalic mesenchymal cells which mainly contribute to vascular cells.

### Contribution of Hlf-expressing cells in the rostral trunk to the tissue vasculature

Since a few Hlf-lineage cells were found in the rostral trunk region, we next examined whether Hlf-lineage cells also contribute to the vasculature of the lung, liver, and heart at E15.5 (Fig. 4E,F). Immunostaining analysis showed a significant contribution of tdTomato-expressing Hlf-lineage cells into the vasculature of lung (5.5±1.2% of blood vessels were positive for tdTomato), but not liver (0.95±0.44%) and heart (2.2±1.5%) in *Hlf-Cre^ERT2^; ROSA-LSL-tdTomato* embryos at E15.5 (Fig. 4G-O,R). These data suggest that Hlf-expressing cells in the rostral trunk at E8.0 are angiogenic mesenchymal cells. Note that some Hlf-expressing cells in the trunk at E8.0 also contribute to non-vascular cells such as cardiomyocytes in the heart (Fig. 4P-Q). Meanwhile, tdTomato-expressing Runx1-lineage cells largely contribute to the vasculature of liver (33.7±3.5% of blood vessels were positive for tdTomato) and heart (12.2±4.7%), but not lung (1.1±0.2%) in E15.5 *Runx1-Cre^ERT2^; ROSA-LSL-tdTomato* embryos (Fig. S4D-L,M). Since Runx1 is expressed by embryonic endothelial cells at E8.0, Runx1-expressing endothelial cells can be a source of the liver vasculature.

### No contribution of Hlf-expressing cephalic mesenchymal cells to tissue-localized macrophages

Having established that cephalic mesenchyme contains hemogenic endothelial cells (Gama Sosa et al., 2021; Li et al., 2012), we next examined the differentiation potential of Hlf- and Runx1-lineage cells into tissue-localized macrophages. No significant contribution of Hlf-lineage cells was found in F4/80^+^ macrophages in brain, liver, lung, and heart in *Hlf-Cre^ERT2^; ROSA-LSL-tdTomato* embryos at E15.5 (Fig. S5A-L,Y). Consistent with previous studies demonstrating that EMPs contribute to tissue-localized macrophages in multiple organs (Gomez Perdiguero et al., 2015; Hoeffel et al., 2015), Runx1-lineage cells contributed to F4/80^+^ macrophages in brain (61.7±12.0% of F4/80^+^ macrophages were positive for tdTomato in the midbrain, 61.0±2.1% in the hindbrain, 64.7±5.4% in the diencephalon), liver (29.0±2.9%), lung (40.4±6.5%), and heart (13.6±6.9%) in E15.5 *Runx1-Cre^ERT2^; ROSA-LSL-tdTomato* embryos (Fig. S5M-X,Y). These data suggest that the differentiation potential of Hlf-expressing mesenchymal cells at E8.0 appears to be restricted to vascular cells, while Runx1-expressing cells at E8.0 can differentiate into both vascular cells and hematopoietic cells including tissue-localized macrophages.

### Hlf-expressing cells at E10.5 are devoid of the angiogenic potential

Previous studies demonstrated that *Hlf* expression marks hematopoietic clusters in the AGM but not EMPs in the yolk sac, and endothelial cells of the yolk sac, AGM, and fetal liver at E10.5 (Yokomizo et al., 2019), but the studies did not examine what *Hlf* expression marks in the head. We first examined whether Hlf-expressing cells at E10.5 have an angiogenic potential. We induced tdTomato expression in *Hlf-Cre^ERT2^; ROSA-LSL-tdTomato* embryos at E10.5 and analyzed the distribution of tdTomato-expressing Hlf-lineage cells at E15.5 (Fig. S6A). Given that brain microglia are mainly derived from yolk-sac EMPs but not AGM hematopoietic progenitors (Hoeffel et al., 2015), Hlf-lineage cells hardly contributed to microglia in the brain parenchymal region (Fig. S6B-J,L): a few Hlf-lineage cells contributed to F4/80^+^ meningeal macrophages (Fig. S6C,F, open arrowheads). Likewise, Hlf-lineage cells were not detectable in the developing brain vasculature (Fig. S6B-J,K). The observation that Hlf-expressing cells at E8.0 are angiogenic but the cells at E10.5 are devoid of the angiogenic potential suggest that a transient expression of *Hlf* marks angiogenic mesenchymal cells.

## DISCUSSION

**Fig. 5.**
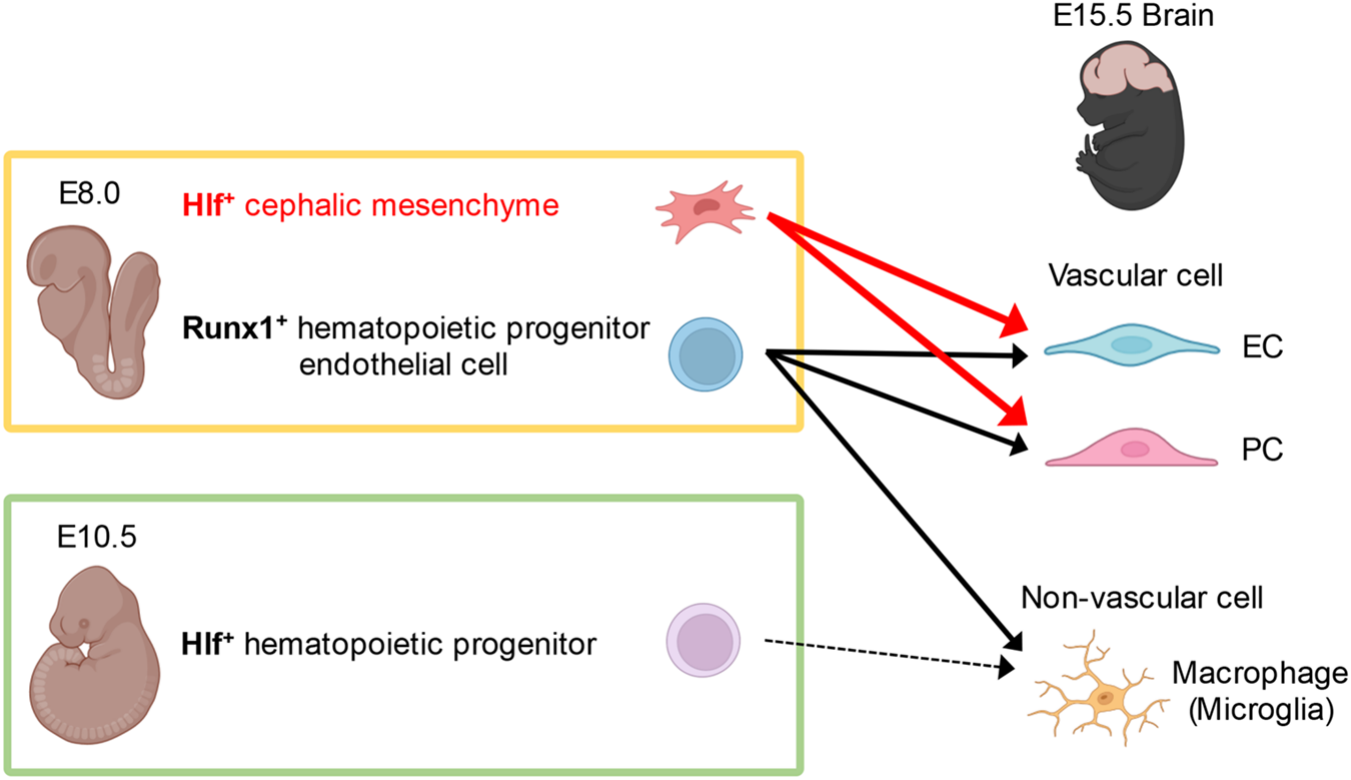
**Contribution of Hlf-expressing cephalic mesenchyme to the brain vasculature.** Graphical summary for the contribution of Hlf-expressing cephalic mesenchymal cells. Hlf-expressing cephalic mesenchyme at E8.0 mainly contributes to brain vascular cells, whereas Runx1-expressing hematopoietic and endothelial progenitors contribute to both brain vascular cells and macrophages. The differentiation potential of Hlf-expressing cephalic mesenchymal cells at E8.0 is restricted to the developing brain vasculature. Hlf marks angiogenic cephalic mesenchymal cells at E8.0, while Hlf-expressing cells at E10.5 are devoid of the angiogenic potential and its expression is restricted to hematopoietic stem cells and progenitors. The graphical summary was created with BioRender.com.

With the genetic lineage tracing experiments using *Hlf-Cre^ERT2^; ROSA-LSL-tdTomato* mouse model, the results presented here identify angiogenic cephalic and rostral trunk mesenchymal cells. Previous genetic lineage tracing experiments using paraxial mesoderm-specific *Cre* lines demonstrated that paraxial mesoderm contributes to trunk and limb vasculature, but these experiments did not show the developmental timing of the paraxial mesoderm commitment into vascular lineage. Given that *Hlf* expression marks vascular cells but not muscle cells in the head and neck regions, Hlf-expressing cephalic mesenchymal cells at E8.0 are mainly committed to vascular lineage (Fig. 5).

Given that the recombination efficiency of *Hlf-Cre^ERT2^* is high, based on the observation that the expression of *tdTomato* reporter is present in the vast majority of Hlf-expressing cells in *Hlf-Cre^ERT2^; ROSA-LSL-tdTomato* embryos at E9.0 (24 h after tamoxifen administration) (Fig. S2I-P), the Hlf-lineage contribution in the brain vascular cells at E15.5 is relatively low (Fig. 3V, the contribution of Hlf-lineage cells in the whole-brain vasculature; Fig. 4R, the contribution of Hlf-lineage cells in the vasculature of hindbrain, midbrain, and diencephalon). One potential explanation is that non-Hlf-lineage cells are the major contributors to the brain vascular cells and Hlf-lineage cells merge at some point to contribute to the brain vascular cell populations. Indeed, PECAM-1^+^ endothelial cells in the nascent vascular plexus around the open neural tube (the future PNVP) are negative for tdTomato in *Hlf-Cre^ERT2^; ROSA-LSL-tdTomato* embryos at E9.0 (Fig. 2D), suggesting that Hlf-lineage cells marked at E8.0 do not contribute to these endothelial cells. In this scenario, Hlf marks a subset of angiogenic cephalic mesenchyme cells. Another explanation is that Hlf-expressing cells earlier than E8.0 may initiate the first wave of brain vascularization including PNVP: these Hlf-expressing cells turn off the *Hlf* expression by E8.0, so these cells are not genetically marked in *Hlf-Cre^ERT2^; ROSA-LSL-tdTomato* embryos with tamoxifen administration at E8.0. In this scenario, angiogenic cephalic mesenchyme cells are heterogeneous and transiently express *Hlf* at different developmental time points.

Although the transient expression of *Hlf* marks an angiogenic mesenchyme, it is not clear whether *Hlf* is required for the differentiation of mesenchymal cells into vascular cells. Previous studies demonstrated that *Hlf* homozygous mutants are morphologically normal and fertile (Gachon et al., 2004). Given that *Hlf* is specifically expressed in HSCs in the AGM and fetal liver (Yokomizo et al., 2019) and the adult bone marrow (Komorowska et al., 2017; Wahlestedt et al., 2017), *Hlf* is dispensable for HSC generation. Likewise, given that *Hlf* marks a classical mesodermal origin of CNS vascular cells, *Hlf* appears not to be required for CNS vascular development. It is possible that other Par-bZip transcription factors (Dbp and Tef), which share a similar DNA binding motif with Hlf, may compensate for loss of *Hlf* in the vascular development. What controls *Hlf* expression may provide an insight in understanding the transcriptional machinery of mesodermal differentiation into vascular cells.

## MATERIALS AND METHODS

### Mice

All animal procedures were approved by the Animal Care and Use Committee of Kumamoto University; the National Heart, Lung, and Blood Institute (NHLBI) Animal Care and Use Committee in accordance with NIH research guidelines for the care and use of laboratory animals. The following mice were used in this study: *Hlf-tdTomato* knock-in mice (Yokomizo et al., 2019), *Runx1-Cre^ERT2^* transgenic mice (Matsuo et al., 2017), and *ROSA-LSL-tdTomato* mice (Madisen et al., 2010). The targeting strategy of *Hlf-Cre^ERT2^* is the same as the one of *Hlf-tdTomato* reporter mice as described previously (Yokomizo et al., 2019). *Hlf-Cre^ERT2^* knock-in mice were generated in the Kumamoto University by inserting a *T2A-Cre^ERT2^* gene fusion before the endogenous stop codon within exon 4. The targeting construct was knocked into the locus using the CRISPR/Cas9 method. The detailed procedure will be reported elsewhere (Yokomizo et al., *Nature* in press). The *Cre*-mediated excision was induced by administrating 2.5 mg tamoxifen (T-5648, Sigma-Aldrich) by oral gavage at embryonic days (E) 8.0 or 10.5 and embryos were harvested at E9.0 or E15.5. *Hlf-Cre^ERT2^; ROSA-LSL-tdTomato or Runx1-Cre^ERT2^; ROSA-LSL-tdTomato* double heterozygous embryos were used for all experiments.

### Whole-mount immunostaining of embryos

Staining was performed essentially as described previously (Yokomizo et al., 2019, 2012). Staining was performed using anti-PECAM-1/CD31 antibody (Clone MEC 13.3, BD Pharmingen, 1:500) to detect endothelial cells and anti-RFP antibody (Rockland, 1:1000) to detect tdTomato. For immunofluorescent detection, either Cy3 or Alexa-647 conjugated secondary antibodies were used. All confocal microscopy was carried out on an Olympus FV1200 confocal equipped with GaAsP PMT detectors (Olympus).

### Section immunostaining of embryos

Staining was performed essentially as described previously (Li et al., 2013). Embryos were fixed with 4% paraformaldehyde/ PBS at 4°C overnight, sunk in 30% sucrose/ PBS at 4°C and then embedded in OCT compound. Embryos were cryosectioned at 25 μm thickness and collected on pre-cleaned slides (Matsunami, Japan). Staining was performed using Armenian hamster anti-PECAM-1/CD31 antibody (Clone 2H8, Chemicon, 1:200) to detect endothelial cells, Rat anti-PDGFRß antibody (Clone APB5, eBioscience, 1:100) to detect pericytes, Rabbit anti-DsRed antibody (Takara, 1:1000) to detect tdTomato, Rat anti-F4/80 antibody (Clone BM8, Invitrogen, 1:500) to detect macrophages, and Mouse anti-Desmin antibody (Clone D33, Dako, 1:500). For immunofluorescent detection, either Alexa- 488-, Alexa-568-, or Alexa-647 conjugated secondary antibodies (Jackson ImmunoResearch or Thermo Fisher Scientific, 1:250) were used. All confocal microscopy was carried out on a Leica TCS SP5 confocal (Leica). Area of tdTomato positive blood vessels and macrophages were quantified using ImageJ (NIH). The percentage of tdTomato-positive blood vessels was based on the area of tdTomato-positive blood vessels within the area of PECAM-1-positive blood vessels (Fig. 4R; Figs S4M, S6K). Likewise, the percentage of tdTomato-positive macrophages was based on the area of tdTomato-positive macrophages within the area of F4/80-positive macrophages (Figs S5Y, S6L).

### Cell preparation and flow cytometry

Single-cell suspensions of embryonic brains were prepared by treating tissues with 0.1% collagenase/0.3% Dispase/0.05% DNaseI solution for 45 min at 37°C. Cells were stained with fluorescence-conjugated antibodies: CD45-BB700 (Clone 30-F11, BD Biosciences), Ter119-FITC (Clone TER-119, BioLegend), PECAM-1/CD31-BV421 (Clone MEC 13.3, BD Biosciences), and PDGFRß-APC (Clone APB5, eBioscience). Non-staining controls were used to define positive gates. Cells were analyzed by FACS AriaIII (BD Biosciences).
